# The impact of social capital and mental health on medication adherence among older people living with HIV (PLWH)

**DOI:** 10.1186/s12889-021-12251-0

**Published:** 2021-12-11

**Authors:** Lei He, Bin Yu, Jun Yu, Jun Xiong, Yuling Huang, Tian Xie, Qi Chai, Bo Gao, Shujuan Yang

**Affiliations:** 1 West China School of Public Health and West China Fourth Hospital, Sichuan University, Chengdu, 610041 Sichuan China; 2 West China Second University Hospital of Sichuan University and Key Laboratory of Birth Defects and Related Diseases of Women and Children (Sichuan University), Ministry of Education, Chengdu, 610041 Sichuan China; 3 Lu County Center for Disease Control and Prevention, Luzhou, 646100 Sichuan China; 4 Sichuan Center for Disease Control and Prevention, Chengdu, 610041 Sichuan China; 5 Pidu District Center for Disease Control and Prevention, Chengdu, 611730 Sichuan China

**Keywords:** HIV, Social capital, Mental health, Medication adherence

## Abstract

**Background:**

The number of older people living with HIV (PLWH) is increasing. Although there are many studies affecting medication adherence, research on the impact of social capital and mental health on medication adherence in this particular population is limited.

**Method:**

Data were collected from an ongoing observational prospective cohort study, starting from November 2018, among older PLWH in Sichuan province, China. Five hundred twenty-one participants were interviewed. Social capital consists of the individual and family (IF) scale, and the community and society (CS) scale. The presence of probable depression and probable anxiety were assessed using the CES-D-10 and GAD-7 scales. Adherence was defined as taking ≥80% of prescribed HIV medication in 4 days prior to the interview. Two sets of Firth’ penalized regression analyses were used to estimate the association between social capital, mental health, and medication adherence.

**Results:**

The prevalence of non-adherence was 18.2% (95/521) among older PLWH in this study. After adjusting for significant factors, the CS social capital (OR: 0.92, 95%CI:0.85–0.99, *p* < 0.05) and probable anxiety (OR:1.73, 95%CI:1.07–2.80, *p* < 0.05) were associated with non-adherence.

**Conclusion:**

This study highlighted that the effects of social capital and mental health on older PLWH’s adherence, which implied that the need to develop interventions to concern for mental health and enhance CS social capital to help the older PLWH better manage HIV medication adherence.

## Background

According to Global HIV&AIDS statistics-2019 fact sheet, there were 37.9 million people living with HIV. After the introduction of antiretroviral therapy (ART), a combination of several antiretroviral drugs used to treat retroviruses (primarily HIV), there is a significant reduction in opportunistic infections that occurred among PLWH, and life expectancy was prolonged [1]. Previous studies generally define older people living with HIV as HIV-infected people who are aged 50 and over [2, 3]. In China, the average HIV prevalence from 2010 to 2018 among people aged ≥50 years was 1.68%, which was higher than that among the entire population by 0.06% [4]. This is related to the increase of reported cases aged ≥50 years in recent years in China. Low condom use was quite common among older adults, which increased the risk of HIV infection [5]. And most older people have poor knowledge of HIV prevention and lack the corresponding awareness of risk prevention [6]. As one of the high HIV epidemic areas in China [7], the number of reported cases in Sichuan province aged ≥50 years accounted for a rapid increase in the total number of reported cases in Sichuan province during the same period, from 21.83% in 2012 to 42.98% in 2017 [8].

However, for ART to be successful, adherence is crucial, which is strictly associated with virologic suppression that means untransmittable and less at risk for health complications. A meta-analysis of 29 studies indicated that the higher levels of adherence were associated with greater viral suppression [9]. In China, primary care institutions assume a greater responsibility for regular medication distribution and follow-up for PLWH [10]. There is a dedicated staff for PLWH, specially, and PLWH come to the community once a month to receive their medication and voluntary CD-4 testing, which help manage the medication regimen for newly diagnosed with HIV people more effectively. Existing studies indicated that many factors can affect adherence, such as self-efficacy, side effects, social support, and sedentary lifestyle [11–15]. However, the factors associated with adherence among older PLWH may differ from those of younger PLWH. The impact of non-AIDS-related comorbidities (medical, surgical, and mental health) expands with age [3, 16], while adherence increased with lower comorbidity and increased number of medications [17].

Mental health problems such as depression and anxiety are considered as the most commonly non-AIDS-related comorbidities among PLWH [18]. Older PLWH may face more complex physical health and psychosocial challenges than young PLWH as they experience the dual impact of aging and HIV [19]. Nevertheless, mental health has been extensively confirmed to have an impact on adherence. A regional subgroup analysis demonstrated that reported anxiety was positively associated with lower levels of ART adherence in Asia [20]. However, previous studies of mental health and medication adherence have rarely focused on older people.

Social capital, as a social network based on trust, reciprocity and mutual help, is generally defined as a collection of the actual or potential resources which embedded in the social fabric [21]. Some studies have shown that participants increase their medication-taking adherence through access to social capital resources (i.e., family, friends, or providers) [22, 23]. Patients’ social capital can protect them from the risk of non-adherence by helping them access scarce resources, and providing them with material and financial support to improve adherence during treatment [24, 25]. China has experienced rapid population aging and urbanization, along with transition in China’s traditional family-based aging care system [26]. The decrease in family size and migration of young adults have diminished families’ functions in supporting their older members. Older people with low social engagement (manifested in China by low employment rates) and trust are more likely to be neglected, leading to greater stress and health effects [27]. This suggests that particular attention should be given to the role played by social capital of the older as a specific group.

To the best of our knowledge, no study has been conducted to evaluate the impact of social capital and mental health on medication adherence among older PLWH in China. Thus, it is urgent to fill the knowledge gap for providing information of formulating targeted intervention measures and helping the older PLWH better manage HIV medication adherence.

This study, using data from two districts/counties of Sichuan Province that show a high prevalence of HIV, examines the social capital and mental health in explaining variation in medication adherence among older PLWH. This study hypothesizes probable depression, probable anxiety, and weak social capital contribute to poor adherence among older PLWH. Drawing from the literature on the buffering role of social capital [28, 29], we propose another possible hypothesis that social capital may modify the association between mental health and medication adherence.

## Methods

### Study design

This study used a baseline data collected from an ongoing observational prospective cohort study, starting from 2018, among older PLWH in Sichuan province, China (the Sichuan Older HIV-infected Cohort Study, SOHICS). The stratified multi-stage cluster sampling method was described in previous studies [30]. The inclusion criteria for participants were 1) diagnosed with HIV, 2) 50 years and over at the time of diagnosis, 3) residing in Sichuan for at least 5 years, and 4) accessing local treatment.

Medical staff in township health centers screened participants for eligibility and briefly introduced the study. After obtaining written informed consent, participants completed the survey in private rooms of their local health centers. Ethics approval was obtained from the Ethics Committee West China School of Public Health and West China Fourth Hospital, Sichuan University. More details on sampling procedures are described previously [30].

### Measures

#### Evaluation of mental health

Depression and anxiety are considered as the two main types of mental health problems in older PLWH [18]. The Center for Epidemiologic Studies Depression (CES-D)-10 scale, consisting of ten questions on the basis of self-report, was used to measure depressive symptoms in this study. The scale has a high sensitivity and specificity for screening major depression in older adults [31]. The 10-item CES-D among HIV-positive people has been validated with an internal reliability of 0.88 and a 0.83 Cronbach’s alpha [32]. The participants indicated how they felt over the past week with symptoms associated with depression on a 4-point Likert scale (0-rarely or none of the time to 3-most or all of the time). The total score ranged from 0 to 30. Higher scores indicated more severe depressive symptoms. A score above 10 was considered as the presence of probable depression, which has been validated previously [32].

Anxiety symptoms were measured by the Generalized Anxiety Disorder Scale (GAD-7), with a 4-point Likert scale ranging from 0 (not at all) to 3 (nearly every day), which showed the frequency of participants suffering from seven problems over the past 2 weeks. Cronbach’s alpha of this scale was 0.92 in this study. The total score ranged from 0 to 21, with higher scores indicating more severe anxiety symptoms. A score above 5 was defined as the presence of probable anxiety [33].

#### Social capital

Social capital was measured with two scales of a validated Chinese version of Health-related Social Capital Measurement, the individual and family (IF) social capital scale and the community and society (CS) social capital scale, which have been evaluated in previous studies [30, 34]. Both have seven items, which are used to create the total score. The IF scale covers the respondent’s individual social network, interpersonal/family trust, and interpersonal/family support, e.g., “You have many close contacts.” “You always trust people who have social interaction with you.” The CS scale relates to the respondent’s community/society participation, community/society support and community/society trust, e.g., “You frequently participated in activities organized by community organizations in the last year.” “You frequently participated in activities organized by community organizations in the last year.” Participants were asked to rate items on a scale of 1 (strongly disagree) to 4 (strongly agree), and higher scores indicated higher social capital. Both of these scales were validated among old PLWH, which Cronbach’s alphas were 0.638 and 0.657 [30].

### Medication adherence

Adherence was assessed via items adapted from the ACTG adherence questionnaire. Participants were asked to report the missed dose of each medication per day, during the past 4 days. In addition, they were asked when they last missed the medications (past week, past 1–2 weeks, past month, past 1–3 months, > 3 months ago) as a measure of “long-term adherence”.

Adherence for the last 4-days was calculated as the number of doses taken in the last 4 days out of the total number of prescribed doses for those days. With the improved tolerability and enhanced pharmacokinetic properties of modern HIV medications, clinical evidence has found lower adherence levels at 80, 90% are not significantly different from the previous gold standard of 95% in achieving viral suppression [35–37]. Previous research indicated that ART adherence of 80% was the threshold for achieving viral suppression [37]. Thus, participants who reported an intake of ≥80% of prescribed medication were considered adhering and those with a reported intake of < 80% were classified as non-adherence in this study.

### Covariates

Participants provided information on their demographic and HIV-related characteristics including age, sex, place of resident, marital status, monthly income, having a spouse living with HIV, duration on antiretroviral therapy, years since HIV diagnosis, comorbidities other than HIV, and daily medication frequency.

### Statistical analysis

In the analysis of data, t-test was used for continuous variables, while Pearson Chi-square was used for categorical variables, to explore the differences in demographic characteristics, HIV-related factors, social capital, and probable depression and anxiety on medication adherence. Considering the low non-adherence of this study, and to avoid problems in model fitting due to data separation, the influence factors of medication adherence were studied using two sets of Firth’ penalized regression models. Medication adherence served as an outcome variable with 0 = adherence and 1 = non-adherence. The first set of models examined the effects of IF social capital, CS social capital, probable depression, probable anxiety, age and explanatory variables which were associated with the medication adherence in bivariate analysis with a *P*-value of 0.05. In model 2, the interaction of CS social capital with probable anxiety was considered. All statistics were performed using the Statistical Package for the Social Sciences-26.0 for Windows (SPSS, Inc., Chicago, IL, the United States) and The R Project for Statistical Computing-4.1.0 for Windows, with *p* < 0.05 considered as statistically significant.

## Results

### Description of the participants

After excluding eight from respondents who had never received antiretroviral therapy, 521 older PLWH completed the study procedures, of which 95(18.2%) indicated non-adherence. 70.8% of them were male, and 56.0% were less than 65 (Table 1). Over half of participants was registered permanent residence in rural area (77.4%), married (65.8%). 68.7% of participants has a monthly income of less than 2000 yuan, which is at the lower income level in China. HIV-related characteristics showed that 58.8% had a spouse living without HIV, and more than half of participants were diagnosed with HIV more than 1 year ago (70.9%). 83.9% had a co-comorbidity (e.g., hypertension, tuberculosis, hepatitis). Bivariate analysis showed that having a spouse living with HIV and daily medication frequency were found to be the influential factors for medication adherence (*p* < 0.05).

**Table 1:** Characteristics of the participants

	All (%)	Adherence (%) *n* = 426(81.8)	Non-adherence (%) *n* = 95(18.2)	χ^2^
Demographic characteristics				
Sex				
Male	369 (70.8)	309 (72.5)	60 (63.2)	3.306
Female	152 (29.2)	117 (27.5)	35 (36.8)	
Age (years)				
50–64	292 (56.0)	241 (56.6)	51 (53.7)	0.263
≥ 65	229 (43.0)	185 (43.4)	44 (46.3)	
Place of resident				
Rural	403 (77.4)	327 (76.8)	76 (80.0)	0.465
Urban	108 (22.6)	99 (23.2)	19 (20.0)	
Marital status				
Single	28 (5.4)	23 (5.4)	5 (5.3)	0.012
Married	343 (65.8)	280 (65.7)	63 (66.3)	
Divorced/Widowed	150 (28.8)	123 (28.9)	27 (28.4)	
Income per month (yuan)				
None	39 (7.5)	32 (7.5)	7 (7.4)	2.788
< 2000	360 (68.7)	288 (67.8)	72 (75.8)	
≥ 2000	121 (23.3)	105 (24.7)	16 (16.8)	
HIV-specific information				
Having an HIV-infected spouse				
Yes	201 (41.2)	156 (39.1)	45 (50.6)	3.948*
No	287 (58.8)	243 (60.9)	44 (49.4)	
Duration on antiretroviral therapy (years)				
≤ 2	342 (65.6)	277 (65.0)	65 (68.4)	0.398
> 2	179 (34.4)	149 (35.0)	30 (31.6)	
Years since HIV diagnosis				
< 1	152 (29.2)	128 (30.0)	24 (25.3)	1.085
1–3	204 (39.2)	163 (38.3)	41 (43.2)	
> 3	165 (31.7)	135 (31.7)	30 (31.6)	
Comorbidities other than HIV				
Yes	437 (83.9)	354 (83.1)	83 (87.4)	1.047
No	84 (16.1)	72 (16.9)	12 (12.6)	
Daily medication frequency				
1 time	423 (81.2)	359 (84.3)	64 (67.4)	14.534*
2 times	98 (18.8)	67 (15.7)	31 (32.6)	

*p < 0.05

### Association of medication adherence with social capital, mental health

In bivariate analysis, CS social capital and probable anxiety were found to be the influential factors for medication adherence (*p* < 0.05) (Table 2). Compared to participants who were adherent to medication, the non-adherence was more likely to have probable anxiety (43.2% vs. 29.1%, *p* < 0.05) and had lower CS social capital scores (*p* < 0.05) (Table 2).

**Table 2:** Association of medication adherence with social capital, probable depression and probable anxiety of the participants

	All (M ± SD/%)	Adherence (M ± SD/%)	Non-adherence (M ± SD/%)	*P*
IF social capital	19.2 ± 4.9	19.3 ± 4.9	18.8 ± 4.6	0.231
CS social capital	23.9 ± 3.0	24.0 ± 3.1	23.3 ± 2.5	0.013
Probable depression				
Yes	171 (32.8)	132 (31.0)	39 (41.1)	0.059
No	350 (67.2)	294 (69.0)	56 (58.9)	
Probable anxiety				
Yes	165 (31.7)	124 (29.1)	41 (43.2)	0.008
No	356 (68.3)	302 (70.9)	54 (56.8)	

*IF* Individual and family, *CS* Community and society

Table 3 showed the association between social capital, mental health, and medication adherence. In model 1, after adjusting having a spouse living with HIV, daily medication frequency which revealed *P* < 0.05 in bivariate analysis and age, probable anxiety was significantly associated with non-adherence (OR = 1.73, 95%CI:1.07–2.80). The CS social capital was positively associated with medication adherence (OR = 0.92, 95%CI:0.85–0.99). The finding also suggested that the risk of non-adherence was 2.69 times higher for 2 times daily medication frequency compared to once a day. Model 2 indicated that the interaction between CS social capital and probable anxiety was no longer associated with medication adherence, nor was CS social capital and probable anxiety interaction term associated with medication adherence (*p* = 0.73). The same effect as in model 1 was found for daily medication frequency (OR = 2.68, 95%CI:1.58–4.50).

**Table 3:** Penalized logistic models for association of social capital, mental health to medication adherence

Non-adherence		
OR (95%CI)		
	Model 1	Model 2
IF social capital	–	–
CS social capital	0.92* (0.85–0.99)	0.91 (0.81–1.01)
Probable anxiety	1.73* (1.07–2.80)	0.91 (0.02–35.55)
Probable depression	–	–
Age	1.24 (0.77–1.98)	1.24 (0.77–1.99)
Having an HIV-infected spouse	0.67 (0.41–1.07)	0.66 (0.41–1.06)
Daily medication frequency,	2.69* (1.58–4.51)	2.68* (1.58–4.50)
CS social capital*probable anxiety	–	1.03 (0.88–1.20)

Model 1 included CS social capital, probable anxiety, additionally adjusted age, having an HIV-infected spouse and daily medication frequency

Model 2 further considered the interaction of social capital and mental health (CS social capital*probable anxiety)

*IF* Individual and family, *CS* Community and society

*p < 0.05

## Discussion

Although there is no cure for HIV, antiretroviral therapy overcomes the adverse consequences associated with the infection [38], leading to a significant reduction in HIV/AIDS-related mortality [39, 40]. The issue of medication adherence has become a widespread concern topic. This study examined the impact of social capital and mental health on medication adherence among older PLWH. The study finding showed that 18.2% of older PLWH demonstrated non-adherence, which is lower than that of other developed countries [11, 16]. This may be related to the management model of HIV in China. The AIDS Prevention and Control Ordinance states free antiretroviral medication is available to patients who are economically disadvantaged in China, which may contribute to older PLWH who have lost the creativity of their wealth to maintain high medication adherence in face of expensive medication prices [38].

The regression model used in this study indicated that higher risk of non-adherence occurred with the weak CS social capital. Although traditional family social capital remains the most important source for older people in Chinese society, Chinese family structure, and intergenerational exchanges have undergone great transitions [41]. The role of community and society-based trust and interaction is gradually growing [26]. Higher levels of community interaction and social trust may facilitate the breadth and depth of the spread of positive thoughts [42, 43], which may allow patients to maintain a positive mindset and behavior to cope with HIV, as well as facilitate the management of older people living with HIV by primary care providers. According to Berkman et al. framework, social networks might operate at the behavioral level via social support [44]. Compared to the young PLWH, the older PLWH usually confronts less social support from friends or families in China [45]. The older PLWH were less likely to disclose their HIV status to family, friends and relatives [46]. As the life course changes, older people are faced with decrease in property, loss of spouse and increase chance of illnesses. All of these may limit their social network with the community and society, which affect older people’s access for support of their medication taking behaviors.

In addition, probable anxiety showed an effect on adherence. This result was consistent with other studies which reported poor adherence was associated with anxiety disorder [47, 48]. With less knowledge of the disease, newly diagnosed older people are more likely to fall into extreme panic for fear of infecting family members, fear of not being able to treat the disease, and fear of losing their reputation among relatives or neighbors [49]. Thus, new infections in aging may affect the role of anxiety on poorer adherence. This feeling is likely to prevent them from being actively consulted and testing. More psychological attention should be given to the newly diagnosed old people.

Considering existing studies suggested that mental health and social capital were interconnected [30], the interaction of probable anxiety and CS social capital were taken into account in this study, but there was no significant association between interaction term and medication adherence.

Meanwhile, from the regression model, factors regarding treatment situation may play an important role. The result showed that daily medication frequency had an effect on adherence, which still remained when controlling for social capital and mental health. There have been studies showing a relationship between the required frequency of medication and adherence [50–52]. Similar to these studies, this study showed that patients taking medication twice daily were more likely to be non-adhering. Four-fifths of the older people in this study had comorbidities other than HIV, and multi-frequency medication use was more likely to bring about non-adherence when faced with the need for medication for other diseases.

The finding suggested that, with an understanding of the psychological needs of older PLWH, leveraging community and society social capital is an effective way to conduct sustainable care among older PLWH. On the one hand, the emphasis on community-based long-term support for HIV/ADIS, with proximity for PLWH to community services, may contribute to better adherence. Primary health care workers can adopt social capital scale tailored to the actual situation in their communities to screen patients, and develop social organizations to provide adequate opportunities for older PLWH to sustain their social involvement. On the other hand, when managing patients, primary health care workers should concern with the psychological changes of elderly patients newly diagnosed with HIV, strengthen the scientific publicity and education of the disease, and cultivate a positive attitude towards treatment, so as to promote their medication adherence.

Some limitations should be mentioned. First, the use of cross-sectional baseline data limited this study to make causal inferences of social capital and mental health on medication adherence. Second, the social capital, such as the retirement pension system and medical insurance system, could be added to fully assess the impact of social capital on adherence among older PLWH. Third, data on medication adherence were gathered through self-report, which is considered to contribute to a recall bias [53]. However, tablet count methods were used to confirm the medication adherence, which could greatly reduce this bias. Further studies using electronic drug monitoring (EDM) to help medication adherence recording should be considered [54]. Finally, the sample was recruited in areas with high HIV prevalence, which should be cautiously considered when generalizing the findings to other regions. In addition, future studies could recruit participants with greater variations from a wider area, which will contribute a more comprehensive knowledge of impact of social capital and mental health on medication adherence.

## Conclusions

This study analyzed the impact of social capital and mental health on medication adherence among older PLWH. The results showed that the CS (community and society) social capital and probable anxiety had an impact on medication adherence. This implied that the need to develop interventions to concern for mental health and enhance community and society social capital to help the older PLWH better manage HIV medication adherence.

## Data Availability

The datasets are available from the corresponding authors on reasonable request.

## Abbreviations

PLWH: People living with HIV
ART: Antiretroviral therapy
IF: Individual and family
CS: Community and society
SOHICS: The Sichuan Older HIV-Infected Cohort Study
CES-D-10: The Center for Epidemiologic Studies Depression-10 scale
GAD-7: The Generalized Anxiety Disorder Scale
